# The Pathogenesis of Tabes Dorsalis

**Published:** 1911-02

**Authors:** T. A. Williams

**Affiliations:** Washington, D. C.; Neurologist to Epiphany Dispensary; Member Corresp. Loc. de Neurol. de Paris; Loc. de Psychol. de Paris; Memb. Assoc. Soc. Clin. de Med. Ment., Etc.


### THE PATHOGENESIS OF TABES DORSALIS.

By Dr. T. A. Williams, Washington, D. C.

*Neurologist to Epiphany Dispensary; Member Corresp. Loc. de Neurol. de Paris; Loc. de Psychol. de Paris; Memb. Assoc. Soc. Clin. de Med. Ment., Etc.*

(*Continued from January Issue.*)

I do not, however, wish to exclude the primary peripheral origin of the nerve degeneration in some tabetics, for the infection at the root of tabes does not seem to be confined to the neural canal, as is shown by recent researches, which has shown the commonness of grave interstitial change in the liver, speen and other viscera.

Strong objections to the pathogenesis advocated in this paper have recently been made by Beutler (78), on account of the very marked vascular engorgement he found developing in the posterior columns in 14 cases, most carefully examined. He believes this too great to be accounted for by the meningitis always present. But it is not unusual to find vascular engorgement of neural tissue undergoing degeneration, and, as previously pointed out, the meningeal lesions tend all the time to regress and leave little trace. As pointed out by Paviot (79), they are often destroyed in the post-mortem manipulation.

The transitions between frank tabes, general paralysis, and cerebro-spinal syphilis are shown by such cases as the six of Guillian and Tahon (77), and of the two Camp (76), where decided meningitis of the cord and vascular syphilitic lesions accompanied the tabetic processes. It is unfortunate that in the case of the latter the radicular nerves had not been examined. It must not be forgotten that Nageotte and Babinski contend for the primary meningeal location of the disease, and they believe the lymphocytosis to be sufficiently presumptive of this.

Marie and Guillian (48), although they suppose the affection to be purely syphilitic, think that some peculiarity of the lymph circulation in the posterior columns confines the disease to that region. They do not attempt to explain the lesions on the posterior root, for Guillain's (49), experiments only show that the lymph circulation is from the pia towards the central canal.

The experiments of Orr and Rows (1), further show a circulation of lymph from periphera nerve via spinal ganglion, as well as anterior root, into meninges and cord in its whole circumference. These and other considerations led Ford Robertson and Macrae (50), to interpret the tabetic process as a specific one due to a toxin non-syphilitic, originating in diptheroid organisms situated in mucous membranes, and whose activity was rendered possible by the lowering of bodily resistance due to antecedent depressing conditions, of which syphilis is probably the commonest.

That meningeal reactions may be produced in this way is shown by Orr and Rows (1), but as Robertson and Macrae are making further experiments to answer the bacteriological objections made to their contentions, the consideration of this theory must be left to a future occasion.

When the nature of the tabetic process is considered, it is more difficult to steer a clear course. Each in its way (the researches of Marie and Guillain (33), of Nageotte (2), of Orr and Rows 4), and of Ford Robertson and Macrae (34), tend to show that the affection is on of lympathic distribution, but here agreement ceases. For Nageotte, it is a tertiary syphilis of the meninges, and the toxic lymph attacks the nerve root where it makes most intimate and long contact, i. e., at the point where is receives its meningeal sheaths.

The directly syphilitic nature of the process is supported by such facts as those of Brissaud and Oberthur (35), Dorlesus (36), and of Duhot (37), who reported two cases ensuing respectively two and four years after infection. In each of these the process was rapidly and definitely arrested by intensive mercurial treatment. Another significant fact is reported by Dalous (38), as follows: Out of 21 tabetics, presenting pathognomonic tertiary lesions, nine denied knowledge of infection or recollection of secondary symptoms.

But symptoms regarded as pathognominic of tabes are found even in the secondary stage of syphilis, e. g., Buttino (39), examined 70 syphilites from this point of view, mostly during the first year of the disease. He found in a large number of cases, a diminution of a transient nature in the light reflexes. In 16 cases he found an evanescent leucocytosis. Babinski (40), found it in seven out of seventy-four syphilitics of long standing without any nervous symptoms. Dufour (14), examined 611 patients, and found it only in seven, all of whom confessed to syphilis. Charpentier (42), failed to find the sign in 1,100 healthy individuals, while Sulzer (43), found a fleeting Argyle Robertson sign in twelve out of fifty-three syphilitics he examined from this point of view. Dupuy Dutemps (44), and Cestan's research established the fact; and Mantour (45), collected 64 cases of this kind. Ravaut (46) and Beletre found lymphocytoses in 58 out of 138 cases of secondary syphilis. This was often asociated with exaggeration of the knee-reflexes, and both conditions were recovered from under specific treatment. This exaggeration of reflexes in no way modifies the point of view here sustained, or in poli-neuritis, an exaggeration precedes a loss of the deep reflexes.

The association of lymphocytosis and reflex iridoplegia has been insisted upon by Babinski and Nageotte (47), as has also the association of the latter with aoritis, now known as the syndrome of Babinski. Irregularity of the pupil was found 12 times in 80 old syphilitics, 5 of them without tabetic symptoms.

The postulate of Weigert that the disappearance of the noble elements is followed by neuroglia prolipheration has probably been ultilized in the interpretation of such schleroses as are found in loco-motor ataxia, for in a pure secondary degeneraion of the noble element of the posterior columns, due to the excessive pressure of the cerebro-spinal fluid upon the posterior roots in cases of cerebral tumor, LeJonne (3), found no neuroglia proliferation in the fifteen cases examined. Leri (55), proved by measurement also that the apparent decrease of connective tissue wasting of the posterior roots found by Phillippe (36), in advanced cases of tabes. This absence of schlerosis during an inflammation generally regarded as interstitial has been recently remarkably exemplified by Raymond (7), in a case of comparatively acute sclerosis.

Why, then, is there irregularity and seeming anomaly in the other sensory manifestations of tabetics? Is it not evident that in a process so wide-spread and irregular in its distributions, it must necessarily be of unequal incidence upon different portions of the nerve roots? That local peculiarities will determine the inclusion of now one bundle, now another, is well shown in such chronic involvement as Potts' disease, where the research of Fry (5), shows that the same process may pick out and moreover affect unequally, here a band of protopathic fibres, there a band of epicritic; for in his case of cervical Potts' disease, the patches of anaethesis were most irregularly distributed elements in the corpus calosum in senile degeneration of the brain was in reality due merely to their close approximation, from the disappearance of the nerve structures proper. In some cases the corpus calosum was less than half the usual thickness. The diminution in volume in the posterior column in tabes is a pathological fact, and the schlerosis is therefore not h[illegible] plastic, but cicatricial. The same considerations apply [illegible] in location, degree and modality. These may be bundles of epicritic or of protopathic fibres.

One must take into account, too, the errors induced by the exceeding crudeness of the usual clinical investigation of the sensibility, before the researches of Head indicated methods of precision.

The declaration of changes more particularly in thermic sensibilities antedating Head's researches is of small relative value, unless the actual temperatures employed are noted; while the fallacies of the clinical methods in the investigation of touch are by this time familiar to every neurologist. The conclusions of the careful phychometric research of Vaschide (5), the only one of which I am aware, are vitiated by these considerations.

The investigation of the tactile sense by the haphiesthesiometre of Toulouse—Vaschide (52), is in reality one of the sense of deformation, for its principle is the appreciation of weight. This appears in the research itself, of which one of the conclusions is that he trich-aesthesia is less involved in the tabetic process. Now, Head (6), has shown that the trich-aesthesia is a tactile function entirely disassociated from the deep sensibility. Vaschide's research therefore furnishes valuable data when interpreted in the light we have obtained from Head. In this investigation of temperature, of which the amounts are given, the distinction of epicritic fibres is very manifest with regard t[illegible] certain roots.

### Two Kinds of Afferent Fibres.

It is possible that the vulnerability of fibres even depen[illegible] upon the distinction which has been attempted between tho[illegible]e of slender structure myelinating late and subserving the life of relation, passing up the cord by the zones of Lissauer (w[illegible], however, Nageotte believes to be endogenous, and thick ones early surrounded bc myelin, whose function is that of int[illegible]al tacility, including the conscious notion of attitudes presided over by the cerebellum. Bonnier (53), declares that he v[illegible]tibular nerve is the homologue of the latter posterior fibres, and that a predilection of tabes for this nerve as against the cochlear portion, homologous with the finer fibres, is thus accounted for. As Head (9), has shown, it appears that a single peripheral fibre may convey an impulse which in the cord may select either of these paths.

Were these latter functions always attacked concomitantly, it would be a conclusive proof that the process originated outside the central nervous system, for these two functions follow different paths in the spinal cord, the impulses which go to the cerebrum reaching there homolaterally via the posterior column and heterolaterally via the lemniscous, the optic thalamus, and the internal capsule, while the cerebellum receives its information homolaterally via the lateral columns, the restiform body and the peduncle, and also heterolaterally via part of Gowers tract and in the main the superior peduncle.

The situation of these cerebellar tracts at the periphery of the cord causes a suspicion that they may be peculiarly susceptible to toxin by extension from the meninges, and may play a greater part in the ataxia of tabetics than on is willing at present to admit, although recent researches of Crouzon (54), and Lejonne (55), show how often they are affected in the mixed schleroses of the aged. But clinical investigation has not yet ascertained whether these functions are in tabes always concomitantly affected. In the periphery, however, a single path appears to convey all impulses subserving sense of attitude, whether these ultimately become conscious or not (Head (9).

VESTIBULAR ATAXIA.

Pierre Bonnier (56), lays great stress upon the vestibular causation of much of the ataxia of tabes. Though it cannot be said that his contention had been seriously examined by others in the light of careful clinical observation, yet it does not seem that clinical study, imperfect as it is, could not have failed to distinguish between kinaesthetic ataxia and that of vestibular origin. Nor is all discrimination of vestibular source.

This is proved even in the cephalic segment by the vertical stick illusion, in which, with eyes closed and head inclined, a [illegible]asped vertical rod appears to incline away from the side to which the head leans, and this phenomenon is quite unaffected by the condition of the semi-circular canals or their nerves. It is probably due to kinaesthetic interpretations co-ordinated by experience, and does not differ in the principle of its mechanism from such illusions as the up and down movement of the telegraph wires when passing them in the train, the appearance of the moon passing behind the clouds, when in reality the clouds are crossing the moon, and from the deception of altitudes lent by distance, all these being due to the interpretation of unusual experiences by usual standards.

### BULBAR SYNDROME VERTIGO.

Bonnier (57), refers the ocular-motor palsies to what he terms the bulbar syndrome provoked by irritation of the vestibular nerve and irradiating via Deiter's nucleus towards one or more of the bulbo-contine and menecephalic nuclei from which Deiter's receives fibres, i. e., those of 3, 4, 5, 6, 8, 9, and 10th cranial nerves.

He attributes to this mechanism the tinitus, deafness, giddyness, sudden falls, even without vertiginous sensations, which sometimes occur in tabetics, as well as Romberg's sign and the oculo-motor palsies. He thinks that their baso-phobia is due to a similar overflow into the pneumogastric, producing a bulbar state corresponding to the emotion of terror, and due to what he terms an unconscious vertigo.

He insists that we are no more conscious of the state of absence of vertigo than we are of the absence of thirst, and hence can describe its numerous ways of occurring in only vague terms.

The fact that a patient often falls without any consciousness of vertigo is attributed by Bonnier to the suddenness by which Deiter's nucleus is stimulated by peripheral irritation of the great posterior root, the 8th nerve. He falls, before a nerve impulse reaches the cerebrum and constitutes an impression in consciousness. He compares this fall to that of the epileptic without aura.

Etienne (63), has recently reported a similar case.

It remains to be seen whether these cases really differ from those described by Gowers (58), as vagal attacks, which often appear to be of true epileptic nature. They, however, differ in that consciousness is retained in the latter.

### Optic Atrophy.

The pathological evidences derived from the examination of the optic nerve in tabetics strongly confirm my point of view of the pathogenesis of the tabetic processes.

Originating in the large ganglion cells of the retina these fibers run a long course in close contact with the meninges until they reach their destination in the superior coliculus and the lateral geniculate body, and although they are not homologues of posterior root fibres, but of the arcuate fibres in the medulla and the medium lemniscus in the mid brain, yet they are subjected on account of their situation to the same extension of inflammatory processes upon the meninges as are the posterior roots. Although it has been sustained by Popoff and Von Grosse (59), that the lesions were primitively retinal, on account of the disappearance of the ganglion cells of that membrane, yet the persistence of many cells there, even when the optic nerve is completely atrophied, has been shown by Marie and Leri (60).

This would seem to indicate that the process is not primitively ganglionic. It is right to say however, that Dupuy Dutemps (61), strongly contends that the real cause of the lost light reflex is a diseased condition of the nerve of the iris.

The amaurosis proceeding from the periphery, and the optic atrophy are the clinical concomitants of this peripheric invasion of the optic nerve. The rarity of the commencement of a tabetic amaurosis by a central scotoma is only to be expected when we recollect that he macular bundle runs in the centre of the optic nerve except at the very beginning of its course, where it is peripheral upon the mesial side of the nerve. (Hensohen) (62). It is its evolvement at this spot which accounts for the few cases of central scotoma of tabetic causation, Galezowsky (64).

The central scotoma which ensues upon a hemianopic contraction of the visual field has, however, a different significance, for this is due to the invasion of the macular bundle in the heart of the nerve in the course of an infiltration proceeding from the mesial side, R. Jocos (65), Galezowski (64).

The very bad prognosis in these cases is due to the smallness of the macular bundle and the fact that hemiopic contraction is a proof that this bundle has been reached by the inflammatory process. The bundle in the bandallette described by Marie and Leri (60), which they have always found intact, in common with that of Meynert (65), in cases even of complete optic atrophy has nothing to do with the optic fibres, but runs between the sublenticular region and the basal optic ganglion of Meynert (60). Nageotte 20), believes that the Zonula of Zinn is the usual site of the lesion.

CHANGES IN THE SMYPATHETIC.

It may appear strange that our point of view should be confirmed by those tabetic symptoms which are usually referred to the sympathetic nervous system. It will be recollected that Duchene at one time thought that the lesions found in the cord and rots might be due to hyperaemia induced by a paralysis of the great sympathetic by the tabetic process, and a memorable controversy arose with Vulpian with regard to this. The latter, indeed, found sympathetic lesions only exceptionally, while Charcot (66), in 1888, stated that even in cases which had suffered for years from gastric crises, there was no change in the nerves and gangion of the solarplexus. Raymond (67), in 1894, thought the integrity of the sympathetic was demonstrated.

This was the state of matters when Jean Ch. Roux (68), was inspired in 1900 by Dejerine to investigate the question. He examined seven tabetics and ten bodies dead of other nervous and other diseases. In all the latter he found the sympathetic healthy, while in not one tabetic did he fail to find marked and identical modifications in the sympathetic chain and the great splanchnich.

In both of these and in all the cases he found that the small myelinated fibers were diminished in number, in the chain from 4,440 to about 500, in the splanhnich from about 2,000 to about 1,150 It will be remembered that these myelinated fibers are derived from the spinal cord, certainly from the inter-medio-lateral column, where Bruce (67), found as many as 8,000 characteristic cells, in the grey matter. They are slender fibres possessing a myeline sheath. They leave the cord by the anterior and posterior roots, and they terminate around the sympathetic ganglion cells, from which another fibre takes up the nerve impulse to terminate in muscle and gland. These latter are the so-called fibres of Remak, and possess no myeline sheath. Neither communicate with the posterior root ganglia.

Now, unless Roux's findings are due to an autonomous lesion, they must be secondary to a process in one or both of two regions, i. e., the gray substance of the cord and the spinal roots; and as lesions of the gray substance of the cord have scarely been observed in tabes, this degeneration of the myelinated sympathetic fibres must be due to the root lesions Nageotte has described. That this is the case Roux showed by observing their degeneration in cats after section of the spinal root.

That this is not part of a general peripheral neural degeneration was shown by the conservation of other peripheral nerves, such as the intercostal. The sympathetic gangia, too, were not diseased, nor are any changes presented by the large myelinated fibres which come from the spinal ganglia, as Roux shows these latter were intact in all the cases. Moreover, the diminution of slender myelinated fibers was proportionate to their decrease in the posteris roots in each case.

### Pathogeny of Vescical and Gestive Symptoms.

It is to the destruction of these that Roux attributes the anesthesia of the testicle, bladder, breasts, as well as that of the stomach, which he has especially studied. The lack of the sensory innervation is for him the cause of the vescical hypotonia with its consequent ataxia, to which is due the mictional difficulties of the tabetic. It is to a similar mechanism he attributes the gastric atony, which he believes responsible for much of the ill health, and even of some of the gastric crises occurring in this disease.

Genouville (70), showed that when a tabetic bladder is touched by a sound, a desire to urinate often follows, while this is not the case in normal persons. To a paresthesia of similar mechanism, Roux attributes the gastric crises and segmental referred pain which his observations show to be frequently provoked by dietetic errors or by drugs. The mechanism does not appear to the writer to differ in principle from that in a case he now has under observation where athetoid movements of the toes take place on stimulation of the skin even by exposure to cold.

We may then conclude:

1. That tabes dorsalis is a secondary degeneration in the syphilitic nature.

2. That the arrangement of the meninges surrounding the radicular nerves renders it peculiarly susceptible at that spot to mechanical or toxic injury.

3. That the unequal incidence of the affection upon different fibres of the posterior root is probably due to unascertained peculiarity of structure or arrangement of fasciculi, rather than to any selective toxic influence.

4. That the lesions tend towards resolution and arrest, even though the process may continue during the life of the individual unless topped by specifics.

5. That with this arrest, regeneration tends to occur in the radicular nerve, the amount in the anteror root being relatively considerable, while that in the posterior roots is less in amount and functionally insignificant as a rule.

6. The otherwise inexplicable vaso-motor and cranial nerve symptoms and post-morten findings in this disease are shown to be necessary concomitants of the tabetic process.

7. The question of the pathogenesis of the poly-neuritic manifestations found in tabetics it not yet answered.

### REFERENCES.

Beutler These de Lyon, 1905; Gas. des Hop 1906.

(28). Alquier Lecon. Salpet 1907. P. 62. Les Lesions de la Moelle dans La Maladie de Pott.

Beletre. These de Paris, 1902.

39). Buttino. Riv. di. Pat. Nerv. e Ment. 1906.

(40). Babinski. Rp.

(55.) Babinski. Rev. Neur, 1898.

(29.) Babinski. Do 1901. Soc. Med. des Hop, 1901. Nov.

(47.) Babinski and Nageotte. Soc. de Biol de Paris. 1905.

(56.) Bonnier, Pierre. Le Tabes Labyrinthique Nouv. Icon. Salpet. 1899.

Bonnier, Pierre. Le Sense des Attitudes. Paris.

(53.) Bonnier, Pierre. Le Nerf Labyrinthique Nouv. Icon. Salpet. 1904.

(57.) Bonnier, Pierre. Le Vertige. 1904.

(60.) Bruce. The Intermedio Lateral Tract. Review of Neurology and Psychiatry. 1907. P 1.

Brisand. Congres de Lille. 1906.

(35.) Ebertthur.

Buetler. These de Lyon. 1906.

(82.) Cajal. Trabajos. 1905.

(75.) Camp.

(46.) Camp. The difficulty of diagnosis between Cerebro-spinal Syphilis Tabes.

(66.) Charcot. Lecons Cliniques. 1888.

(64.) Crouzon. Les Scleroses Combinees de la Moelle. These de Paris. 1899.

(42.) Charpentier. These de Paris, 1899.

(38.) Dalous. Les Lesions specifique dans le Tabes. Revue de Med. 1904.

(5.) Duchenne, I. Arch de Med. 1858.

(41.) Dufour. Relations entres les troubles Pupillaires, la Syphilis, et Tabes et Paralysie General.

(37.) Duhot. Ann de la Polyclin. Brux. 1903.

(6.) Dupuy Detemps. Con. d' Ophthal. de Paris. 1906.

(61.) Dupuy Detemps. Annals Oculists. 1905.

(44.) Dupuy Dutemps and Cestan. Le signe Puillaire d' Argylle-Robertson. Sa valeur Semeio logique, ses relations avec la syphilis. Gaz. des Hop. de Paris. 1901. Dec. 28th.

(36.) d'Orleans. These de Paris. 1906.

(63.) Etienne. Un cas de syndrome de Bonnier. Rev. Neur. 1907. 1025.

(27.) Congres de Lille, 1906.

(2.) Ferrier. Brit. Med. Jr. 1906.

51.) Fry, Frank R. Jr. of Nervous and Mental Diseases. 1907. 185.

Galezowski, Jean. These de Paris. 1904. Le fond de l'oeil dans les affections des Systems Nerveux.

(64.) Galezowski et Lobel. Extrait du Recuil d' Opthalmologie. April, 1906. Atrophie Optic Tabetique et Scotome Centrale.

(79.) Genouville. These de Paris. 1894.

(58.) Gowers. Vagal Attacks. 1907. Lancet. June, 1907.

(59. Von Grosz. Congress Internat. d' Opthal. Utrich. 1889.

(49.) Guillain. Le circulation de la Lymphe dans la Moelle Epiniere. Rev. Neur. 1899. Dec.

(77.) Guillain et Tahon. Soc. Biol. Jan., 1905.

(62.) Klinische und Anatomische Beitrage zur Pathologie des Gehirns. Zweite Theil Upsala. 1892.

(65.) Jocqs, R. Bul et Memoire Soc. Med de Paris. 1902. March 24.

(83.) Thoster. Tur Phys. des Spinal Ganglion under trophisth. Nerv. path d' Tabes. Leipsig Engeleran. 1904.

(81.) Congress de Geneve. 1907.

Lannois. Nouv. Icon. Salpet. 1905. P. 593.

(22.) Lamy e Millian. Soc. Medical des. Hop. de Paris. 1015. 991.

Leri. Congres de Lille. 1906. Le Cervau Senile. Arch. gen. de Med. 1905. Page 3009.

(20.) Lebar. These de Paris. 1906.

(45.) Mantoux. These de Paris. 1904.

(32.) Marie et Leri. Traite delned. Bonchard-Brissaud.

IX. XIXIV.

(48.) Marie et Guillain. Rev. Neur. 1903. Jan.

Marie et Leri. Rev. Neur. 1905. P. 493. Persistance d' un faiseau intact dans les bandalette Optique apres Aptrophie Complete des Nerfs. Le Faiseau Residuaire de la Bandelette. Le Ganglion Optique Basal et ses connections.

(72.) Marie. Rev. Neur. 1906.

(65.) Meynert. Psychiatry trans. by Sacho. 1887.

(2.) Nageotte. Soc. de Biol. 1908.

(2.) Nageotte. Rev. Neur. 1906.

(2.) Nageotte. Rev. Neur. 1903. Jan. 15th.

(74. Oddo. Rev. Neur. 1906. 587. Page 639.

(79.) Paviot. Soc. Med de Hop. de Lyon. 1905.

(26.) Phillippe. Arch de Neur. 1897. Le Tabes Dorsalis. Paris. 1897.

(46.) Ravaut. Soc. Med. des Hop. de Paris. 1901.

(15.) Raymond. Rev. Neur. 1906. P. P 1882.

(67.) Raymond. Lecons Cliniques. 1894.

(78.) L' Encephale. 1907. P. 225.

(35.) Robertson and Macrae. Rev. of Neur. and Psy. Feb., March and April, 1906. 1904. May, 1905.

(50.) Robertson and Macrae. Brit. Med. Jr. 1905.

(31.) Redlich. Arbeiten ans. dem Institut fur Anatome et physiologic, etc. 1.

(68.) Roux. These de Paris. 1900.

(43.) Sulzer. Ann. de Dermot. et de Syph. 1901. P. 239. dans le Tabes.

(23.) Sherrington. Jr. of Physiology. 1894. Vol. 25.

(71.) Souques. Rev. Neur. 1907. Atrophie Musculaire

Thomas et Hauser. Nouvel Icon. de la Salpet. Soc. Biol. July, 1902 Loc. Cit. 1904.

(30.) Thomas. Rev. Neur. 1906. 573.

(52.) Toulouse, Vaschile. L'Examin des Sujets (dans le Bibliotheque Internationale du Psychologie Experimental de Toulouse.)

(51.) Vaschide. Elude Psychologic de la Sensibilite dans le Tabes.

